# Distinctive Gut Microbiota in Patients with Overweight and Obesity with Dyslipidemia and its Responses to Long-term Orlistat and Ezetimibe Intervention: A Randomized Controlled Open-label Trial

**DOI:** 10.3389/fphar.2021.732541

**Published:** 2021-08-26

**Authors:** Jin Jin, Ruyue Cheng, Yan Ren, Xi Shen, Jiani Wang, Yigui Xue, Huimin Zhang, Xiuhua Jia, Tingting Li, Fang He, Haoming Tian

**Affiliations:** ^1^ Department of Endocrinology, West China Hospital of Sichuan University, Chengdu, China; ^2^ Department of Nutrition and Food Hygiene, West China School of Public Health and West China Fourth Hospital, Sichuan University, Chengdu, China; ^3^ Frontier Medical Service Training Battalion of Army Military Medical University, Xinjiang, China; ^4^ People’s Hospital of Akto County, Xinjiang, China; ^5^ Health Service Center, Akto County, Xinjiang, China

**Keywords:** gut microbiota, SCFAs, orlistat, ezetimibe, obesity, metabolism

## Abstract

This study investigated the gut microbiota and short chain fatty acids (SCFAs) characteristics of subjects with obesity from Xinjiang in northwestern China, a region with a multiethnic culture and characteristic lifestyle, and to explore the potential microbes that respond to a 12-wk medication of orlistat and ezetimibe with a randomized controlled open-label trial manner. The gut microbiota profile of patients with overweight and obesity with dyslipidemia in Xinjiang was distinctive and characterized by enrichment of *Lactobacillus* and the reduction of the diversity and the depletion of *Actinobacteria*, *Bacteroides*, *Bifidobacterium*, and *Bacteroides fragilis*. *Prevotella*-type, *Gemmiger*-type, and *Escherichia/Shigella*-type were the gut microbial patterns of the Xinjiang population. However, the fecal SCFAs levels and enterotypes were similar between healthy individuals and patients. These results indicated that the contribution of the gut microbiota to obesity was highly dependent on geography and dietary habits. Waist circumference, total triglyceride (TG), and fasting blood glucose (FBG) were significantly decreased after orlistat therapy, whereas TG, total cholesterol (TC), and low density lipoprotein cholesterol (LDL-C) were significantly decreased by ezetimibe. Overall, the gut microbiota and their SCFAs metabolites were relatively stable after treatment with the two drugs, with alteration of some low-abundant bacteria, i.e., significantly increased *Proteobacteria* and decreased *Alloprevotella* after orlistat, and increased *Fusobacteria* and *Fusobacterium* after ezetimibe therapy. These results indicated that intestinal malabsorption of dietary fat and cholesterol caused by orlistat and ezetimibe had a limited effect on the overall gut microbial community and their metabolites. Nevertheless, significant correlations between several core microbes that responded to the medications and biochemical data were found; in particular, *Actinomyces* and *Bacteroides* were positively correlated with FBG after orlistat intervention, while *Clostridium XVIII* and *Lachnospiracea incertae sedis* were negatively correlated with TC and LDL-C after ezetimibe intervention, thus indicating their roles in improving glucolipid metabolism in obesity by acting as potential microbial targets.

## Introduction

The prevalence of overweight and obesity has increased worldwide in the past ∼50 yr, reaching pandemic levels. The Global Burden of Disease study reported that, between 1980 and 2013, the proportion of adults with overweight or obesity increased from 28.8 to 36.9% in men, and from 29.8 to 38.0% in women (Ng et al., 2014). A high BMI accounted for 4.0 million deaths globally, more than two-thirds of which were caused by cardiovascular disease (CVD) (Afshin et al., 2017), which is a frequent consequence of overweight or obesity in adults accompanied by dyslipidemia. According to the Report on the Status of Nutrition and Chronic Diseases of Chinese Residents (2015), the prevalence of overweight among Chinese adults was 30.1%, the prevalence of obesity was 11.9%, and the overall prevalence of dyslipidemia was 40.40% in 2012, which represents a substantial increase from the values recorded in 2002 (18.9, 2.9 and 18.6% respectively). The increase in serum cholesterol levels in the population will lead to an increase of approximately 9.2 million cardiovascular events in China from 2010 to 2030 (Moran et al., 2010). Therefore, effective control of overweight and obesity is of great significance for the prevention and control of CVD worldwide, and especially in China.

Gut microbiota have gained great attention for their complex interactions with many diseases of the host, including obesity. As early as 2005, the Gordon research team demonstrated that obesity could alter the gut microbiota ecology and that a significantly increased ratio of *Firmicutes/Bacteroidetes* was an obvious intestinal microbial characteristic of individuals with obesity (Ley et al., 2005; Turnbaugh et al., 2006). Thereafter, increasing evidence has demonstrated that interventions targeting the modulation of gut microbiota and its metabolites (such as probiotics and prebiotics, etc.) are effective and integrative for obesity and its comorbidities (Canfora et al., 2019; Green et al., 2020).

Xinjiang is a multiethnic region with a unique food culture characterized by a high proportion of mutton and low proportion of dietary fiber. Moreover, the genetic background of the Xinjiang population is complicated, with European and Asian characteristics (Feng et al., 2017; Yin et al., 2020). Studies have implied that dietary habits, ethnicity, and geography substantially impact the taxonomic composition of the microbiome (Deschasaux et al., 2018; He et al., 2018; Kolodziejczyk et al., 2019). In particular, the present knowledge on how the composition of the gut microbiome relates to patient health is principally based on investigations of European and North-American populations. Although this knowledge is useful for understanding the microbiota of individuals from such populations, it may not accurately extrapolate to individuals from distinct populations or living in distinct locations, especially in Xinjiang. Therefore, it is crucial to study the characteristics of gut microbiota and their metabolite profiles in the Xinjiang population, as they may help prevent and treat obesity more precisely using microbial targets.

Medications were also suggested to affect the gut microbiota, especially diabetes medications, such as acarbose and metformin (Gu et al., 2017; Wu et al., 2017). Orlistat is a common antiobesity drug that acts by inhibiting the absorption of triglycerides, whereas ezetimibe is a hypolipidemic drug that selectively inhibits the absorption of cholesterol by attaching to the brush border of the villi epithelium of the small intestine (Nakou et al., 2008). Therefore, the administration of either of the two drugs can result in the accumulation of triglycerides and cholesterol in the colon, respectively. Complex carbohydrates that have not been digested by upstream digestive tract enzymes are the main source of nutrients for gut microbes, and these carbohydrates can be fermented by gut microbiota to produce metabolites, such as SCFAs. Our previous study suggested that high-fat diets and high-cholesterol diets could differentially alter gut microbiota and their metabolites, including SCFAs and bile acids (Liang et al., 2021). However, whether these effects are caused by the high dietary lipid/cholesterol content or to the concomitant decrease of carbohydrate intake is unclear. The effects of orlistat and ezetimibe on gut microbiota are poorly understood, and the characteristics of the bacterial populations involved in lipid and cholesterol metabolism are unclear.

Therefore, our present study aimed to investigate the gut microbiota and SCFAs profiles of patients with overweight and obesity with dyslipidemia in Xinjiang and identify the core microbes that responded to orlistat and ezetimibe, thus suggesting the contributions of gut microbiota to host metabolism.

## Materials and Methods

### Study Design

The study was conducted using a randomized, open-label design in subjects with overweight/obesity with dyslipidemia (patients). Patients were randomly assigned to the orlistat or ezetimibe intervention group via computer-generated random numbers. They were asked to take orlistat or ezetimibe for 12 wk. Fasting blood and feces samples were collected both before and after the intervention. Moreover, a cross-sectional subgroup study was conducted by collecting the feces of healthy volunteers and patients who refused to undergo the drug intervention. The study was conducted in accordance with the Declaration of Helsinki and the standards of Chinese clinical studies. All subjects signed an informed consent form after they were fully informed regarding the purpose, process, and potential risk of this study, as well as the right to withdraw from this study with a detailed study procedure. The study was approved by the Medical Ethics Committee of the West China Hospital, Sichuan University (approval number: 2019-066) and the Ethics Committee of The Fifth Affiliated Hospital of Xinjiang Medical University (approval number: XYDWFYKY 2018-01) and registered in the *ClinicalTrials.gov* (NCT number: NCT03884127; https://clinicaltrials.gov/ct2/results?cond=&term=NCT03884127).

### Subjects

All subjects who were aged 26–71 yr and live in Xinjiang for ≥10 yr were recruited from the Akto County Community Health Service Center, Xinjiang, PR China. First, all participants underwent a questionnaire survey about basic information, such as age, ethnicity, history of disease, and use of antibiotics and probiotics, etc. Second, fasting blood samples were collected from all participants and anthropometric and biochemical parameters were assessed to select the patients with overweight or obesity with dyslipidemia in Akto County People’s Hospital, Xinjiang, PR China. In China, the diagnostic criteria for overweight is BMI ≥24 kg/m^2^, whereas that of obesity is BMI ≥28 kg/m^2^, according to the Guidelines on Prevention and Treatment of Obesity in Chinese Adults (Chen and Lu, 2004). The BMI of the participants was calculated using the following equation: BMI = weight (kg)/height^2^ (m^2^). Anthropometric data were collected using standard techniques and included body weight (BW), height, waist circumference (WC), and hip circumference (HC). An automatic biochemical analyzer was used to detect fasting blood glucose (FBG), total triglyceride (TG), total cholesterol (TC), low density lipoprotein cholesterol (LDL-C), high density lipoprotein cholesterol (HDL-C), γ-glutamyl transpeptidase (γ-GT), alanine aminotransferase (ALT), aspartate aminotransferase (AST), and albumin (ALB). The diagnostic criterium for dyslipidemia is TC ≥5.2 mmol/L, or TG ≥1.7 mmol/L, or LDL ≥3.4 mmol/L, or non-HDL-C ≥4.1 mmol/L, according to the Guidelines for the Prevention and Treatment of Dyslipidemia in Chinese Adults (Joint committee for guideline, 2018). Accordingly, subjects whose BMI was 18.5–23.9 kg/m^2^ and whose blood lipids were in the normal range were included in the control group, while subjects who were diagnosed with both overweight/obesity and dyslipidemia were considered as the patient group. Patients who agreed to receive the drug intervention were assigned in a 1:1 ratio to the orlistat and ezetimibe groups based on their own choices. Participants were asked to provide fasting blood and fecal samples.

Exclusion criteria: *1*) history of other endocrine and metabolic diseases or dysfunction, gastrointestinal disease, liver disease, acute and chronic pancreatitis, infectious diseases, and mental disease, or liver and renal function test >2.5 times the upper limit of normal; *2*) long-term use of health-care products and probiotics; *3*) use of antibiotics or other drugs within 6 mo before participating in the study; *4*) alcohol and/or psychoactive substance abuse, or drug abuse or dependence in the past 5 yr; *5*) pregnancy or preparing to become pregnant, or breastfeeding; *6*) allergy to the ingredients of orlistat capsules and ezetimibe tablets; and *7*) need to go out frequently, poor compliance with the medication, etc.

### Sample Size Calculation

First, a sample size calculation was performed as previously described (Gu et al., 2017). PASS 15.0 (Non-Inferiority Tests for the Difference Between Two Means, power = 0.80, alpha = 0.05, NI = N2) was used to calculate the sample size. This calculation was based on an average difference of 0.3 mmol/L fasting cholesterol between the orlistat group and the ezetimibe group at the end of the study. Therefore, the calculated sample size of each group was 20 cases. Second, to increase the biological duplications, the sample size was increased to 30 cases per group at least. Third, assuming that the proportion of loss to follow-up was 20%, the sample size of each group was calculated as 36 cases. Finally, to collect as many qualified fecal samples as possible, the size of each group was increased by 10%. Therefore, the final sample size of each group was 40 cases.

### Drugs

The two drugs used in this study were orlistat capsules and ezetimibe tablets, which have been on the market for more than 5 yr. Detailed safety information about the drugs is available and they were strictly used in this clinical study according to the drug instructions. Orlistat capsules (commercial name, Yasu) (approval number: H20123131; Zeni Pharmaceutical, Chongqing, PR China) have a specification of 0.12 g × 18 capsules. Orlistat capsules were orally administered two to three times per day, one capsule each time, taken with meals or within 1 h after meals; if there was no meal or the food did not contain oil, participants were allowed to omit one capsule. Ezetimibe tablets (commercial name, Ezetrol) (approval number: H2016081; MSD Pharma (Singapore) Pte. Ltd., Gateway West Singapore) have a specification of 10 mg × 5 tablets. Ezetimibe tablets were orally administrated once at night, one tablet at a time. The medical course of the study was 12 wk. All drugs were kept and distributed by the researchers or staff at the Aktao County Community Health Service Center.

After taking the two medicines, the community health service station staff and researchers followed-up the tested patients every 4 wk by inquiring about the number of remaining tablets/capsules, to judge and record the subject’s compliance with the medication. Participants who took any other medicines for treating dyslipidemia or that disturbed the gut microbiota and whose compliance was poor were excluded from the study.

### Fecal Samples Collection and Fecal DNA Extraction

A sterile 50 ml centrifuge tube, a sterile tongue depressor, a disposable paper cup, and disposable gloves were used to collect the fecal samples. The operation sequence was as follows: ①Use a disposable paper cup to save fresh morning feces before or after breakfast; ②Use a sterile tongue depressor to take the middle part of the feces into 50 ml centrifuge tube, then the feces were divided into three tubes, each tube was at least 50 g; ③The feces were transferred to the community health service station and be frozen at −80°C within 1 h. The operating procedures of fecal samples collection were fully introduced to all the subjects before the start of the study and the collection operation was completed by subject itself. Since the feces storage location (community health service station) was very close to the subject’s residence, no fecal preservation liquid was used.

Fecal DNA extraction from 200 mg of feces was processed using a TIANamp Stool DNA Kit (Tiangen Biotech Co. Ltd., Beijing, PR China) and the procedures were operated strictly according to the manufacturer’s instructions.

### 16S rRNA Sequencing and Microbial Diversity and Community Analysis

As described previously (Cheng et al., 2019), briefly, the V3−V4 hypervariable region of fecal bacterial 16S rDNA was amplified, and the PCR products were confirmed by 2% agarose gel electrophoresis and purified. Subsequently, the amplicon library was assessed and sequenced on an Illumina MiSeq instrument (Illumina Inc., CA, United States). First, demultiplexed FASTQ files were generated from base-calls using Illumina’s bcl2fastq (v1.8.4) software, then reads with missing barcodes, incorrect barcodes, or conflicting barcode pairs were discarded and mismatches per primer more than three were cut off using Trimmomatic (v0.36). Second, paired-end reads were merged using FLASH (v1.2.11), and then the sequences were quality filtering by a 10-bp sliding window, followed by a minimum length restriction of 100 bases using Trimmomatic. After this, sequences were defined as high-quality sequences.

Then, chimeric sequences were detected and removed using the USEARCH (v10.0.240_i86linux32) UNOISE3 algorithm. An OTU abundance table was then generated using the otutab command. Taxonomic assignments to the OTUs were performed with the SINTAX algorithm implemented in USEARCH based on the Silva Living Tree Project v123 (LTPv123) database (16S V3-4). Taxonomic assignments were considered reliable when bootstrap confidence values exceeded 0.75. Multiple sequence alignment and phylogenetic tree construction were conducted using the USEARCH cluster_agg command with the default parameters.

Subsequently, based on the OTU table and phylogenetic tree, the alpha diversity index (Observed OTUs, Chao 1, ACE, Fisher, Shannon and Simpson index) was calculated with QIIME (v1.9.1) and an analysis of community structure and species abundance difference at each taxonomic level was performed. The beta diversity was identified by a Partial Least Squares Discrimination Analysis (PLS-DA) based on the family or genus level using Phyloseq package (v1.20.0). PICRUSt (v1.2.1) was used to derive relative KEGG pathway abundance. A supervised analysis was performed using LefSe (Log 10 LDA scores >2) to elicit the microbial functional pathways that were differentially expressed in the different groups. All plots were created using R (v3.4.1). Sequenced Read Archive (SRA) accession number for the raw data of the 16S rRNA sequencing is PRJNA674844 (https://www.ncbi.nlm.nih.gov/sra/PRJNA674844).

### Enterotype Analysis and Weighted Correlation Network Analysis (WGCNA)

Enterotype analysis was processed as reported by Arumugam et al. (Arumugam et al., 2011). Differences in alpha diversity between enterotypes were analyzed using the Wilcoxon test. Individual enterotypes were visualized by heatmap using HemI (v1.0.3.7). WGCNA can be used for identifying clusters (modules) of highly correlated genes, for summarizing such clusters using the module eigengene or an intramodular hub gene, for relating modules to one another and to external sample traits (using eigengene network methodology), and for calculating module membership measures. Correlation networks facilitate network-based gene-screening methods that can be used to identify candidate biomarkers or therapeutic targets (Zhang and Horvath, 2005). In this study, OTUs were considered as being equivalent to genes.

Briefly, datasets were constructed using the following methods. A signed weighted correlation network was constructed by first creating a matrix of pairwise correlations between all pairs of OTUs chosen based on variance. The resulting Pearson’s correlation matrix was transformed into a matrix of connection strengths (e.g., an adjacency matrix) using a power of 3 (Orlistat group) or 4 (Ezetimibe group). Subsequently, the topological overlap was calculated to measure network interconnectedness. Average linkage hierarchical clustering was used to group OTUs on the basis of the topological overlap dissimilarity measure (1-topological overlap) of their network connection strengths. Using a dynamic tree-cutting algorithm and a merging threshold function of 0.25, we identified six modules in the dataset. The freely available statistical analysis software WGCNA package (v1.68) and R v3.4.1 tutorials for constructing a weighted gene coexpression network have been described previously (Langfelder and Horvath, 2008). We summarized the expression profile of each module based on the corresponding module eigengene (i.e., the first principal component obtained by singular value decomposition). We then defined the module membership for each OTU with respect to each module as the Pearson’s correlation between the expression level of the OTUs and the module eigengene, also known as module eigengene-based connectivity (kME). This measure was naturally scaled to lie in the interval of [−1, 1]. OTUs with the greatest module membership values were referred to as intramodular hub OTUs. To identify modules associated with external traits, we first calculated the module eigengenes of each module, then correlated these with the external traits using Pearson’s correlation coefficients, and modules with *p*-values < 0.05 were classified as trait-related modules. The OTUs included in these modules were determined as the core OTUs between groups. These annotated genera were determined as the core microbes that responded to the drug interventions. The correlations between the genera in each module and the SCFAs and biochemical data of the participants were assessed using Spearman’s correlation analysis. Network plots highlighted the correlations between positive and negative responders to SCFAs, between before and after the drug interventions. The lines between nodes represent correlations between the connected nodes, with line width indicating the magnitude of the correlation. R (v.3.4.1) was used for visualization.

### Fecal SCFAs Detection

Gas chromatography–mass spectrometry (GC-MS) Agilent 6890N/5975B (Agilent Technologies, Inc., United States) was used to detect fecal SCFAs levels. Briefly, 100 mg of feces were added to 100 μl of 15% phosphoric acid (China National Pharmaceutical Group Co. Ltd., Beijing, PR China), 100 μl of a 250 μg/ml internal standard (isohexanoic acid; Sigma) solution, and 400 μl of ether (China National Pharmaceutical Group Co. Ltd., Beijing, PR China) and homogenized for 1 min. After centrifugation at 12,000 rpm at 4°C for 10 min, the supernatant was collected and tested on the GC-MS instrument. Acetic acid (AA), propionic acid (PA), isobutyric acid (IBA), butyric acid (BA), isovaleric acid (IVA), and valeric acid (VA) were purchased from Sigma. Caproic acid (CA) was purchased from Aladdin (Aladdin Shanghai Biochemical Technology, Shanghai, PR China).

### Statistical Analysis

SPSS 25.0 was used for statistical analyses. Unpaired Student’s test or the Mann−Whitney *U* test was used to analyze the data in the control and patient groups. Paired Student’s test or the Wilcoxon matched-pairs signed rank test was used to analyze the data before and after drug intervention. The χ^2^ test was used to analyze sex and ethnicity effects. Significance was set at *p* < 0.05. All tests were two tailed.

## Results

### Study Population

**FIGURE 1:**
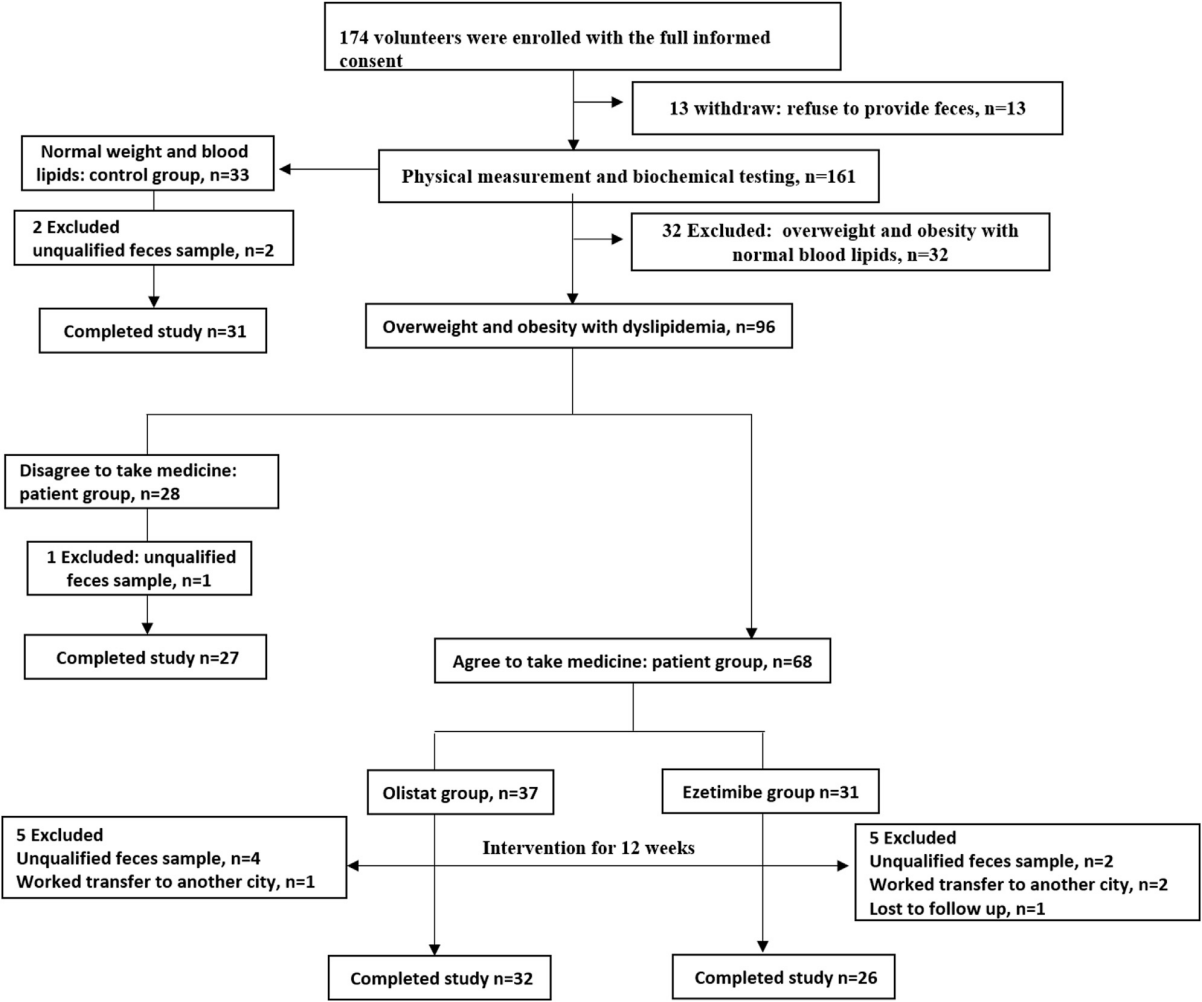
Participants flow in this study. Values are expressed as the number of participants.

The participant flow of this study is shown in Figure 1. A total of 174 volunteers were enrolled in this study after full informed consent was obtained. Thirteen volunteers withdrew from this study because of the refusal to provide fecal samples. According to the strict inclusion and exclusion criteria, 32 volunteers with overweight or obesity were excluded because of normal lipid levels detected after physical and biochemical measurements. Therefore, 33 healthy volunteers were included into the control group, while 96 volunteers were diagnosed as having overweight and obesity with dyslipidemia. Among these patients, 28 individuals who refused to take the medicine intervention were assigned to the patient group, whereas the 68 who agreed to receive the medicine intervention for 12 wk were respectively assigned into the orlistat (*n* = 37) and ezetimibe (*n* = 31) group according to their own choice. During the experiment, two participants in the control group, one in the patient group, five in the orlistat group, and five in the ezetimibe group were excluded for various reasons. Therefore, 31 participants in the control group, 27 in the patient group, 32 in the orlistat group, and 26 in the ezetimibe group completed the whole study. Furthermore, no adverse events associated with the administration of the two drugs were reported. The demographic and baseline characteristics of all the participants are listed in Table 1. All the 85 patients (including patients in patient group and two drug groups before intervention) were overweight or had obesity with significantly increased BW, BMI, WC, and HC, and were diagnosed as having dyslipidemia with significantly increased TG, TC and LDL-C (only found in orlistat and ezetimibe group) and significantly decreased HDL-C, as well as liver dysfunction presenting as increased γ-GT and ALT, and the ALB in orlistat and ezetimibe groups were significantly lower than controls. Moreover, patients in the two drug groups before intervention were significantly older than healthy controls. Studies have reported that the diversity and composition of gut microbiota were age-dependent, which means age will be a confounding factor in the analysis of gut microbiota (de la Cuesta-Zuluaga et al., 2019). Therefore, we separately set up an age-matched “patient group” to compare with control group.

**TABLE 1:** Demographic and baseline characteristics of healthy participants and patients.

Characteristics		Control *n* = 31	Patient *n* = 27	*p*-value	Orlistat *n* = 32	*p*-value	Ezetimibe *n* = 26	*p*-value
Age (yr)		31.581 ± 2.896	33.000 ± 2.481	0.052^a^	40.053 ± 8.107	<0.0001^b^	45.385 ± 9.100	<0.0001^b^
Sex, *n* (%)	Male	18 (58.1%)	17 (62.96%)	0.704^c^	21 (65.6%)	0.537^c^	16 (61.5%)	0.790^c^
	Female	13 (41.9%)	10 (37.04%)		11 (34.4%)		10 (38.5%)	
Ethnicity, *n* (%)	Han	16 (51.6%)	15 (55.56%)	0.764^c^	23 (71.9%)	0.098^c^	10 (38.5%)	0.321^c^
	minority	15 (48.4%)	12 (44.44%)		9 (28.1%)		16 (61.5%)	
Height (cm)		169.290 ± 7.942	170.524 ± 7.527	0.548^a^	166.783 ± 6.382	0.172^a^	166.3654 ± 9.77912	0.218^a^
BW (kg)		58.387 ± 5.841	87.099 ± 17.949	<0.0001^b^	80.574 ± 11.574	<0.0001^a^	84.108 ± 15.508	<0.0001^a^
BMI (kg/m^2^)		20.343 ± 1.100	29.810 ± 5.004	<0.0001^a^	28.938 ± 3.618	<0.0001^a^	30.272 ± 4.117	<0.0001^a^
WC (cm)		75.032 ± 3.582	98.033 ± 13.947	<0.0001^b^	98.604 ± 11.926	<0.0001^b^	101.942 ± 11.935	<0.0001^b^
HC (cm)		88.065 ± 4.242	105.833 ± 9.888	<0.0001^a^	105.059 ± 8.549	<0.0001^a^	107.577 ± 10.221	<0.0001^a^
FBG (mmol/L)		4.632 ± 0.567	4.868 ± 1.151	0.601^b^	5.440 ± 2.306	0.112^b^	5.865 ± 2.194	0.001^b^
TG (mmol/L)		1.088 ± 0.476	2.983 ± 1.411	<0.0001^a^	3.809 ± 2.467	<0.0001^b^	3.578 ± 2.568	<0.0001^b^
TC (mmol/L)		3.935 ± 0.626	4.499 ± 0.899	0.007^a^	4.941 ± 1.718	<0.0001^b^	5.277 ± 0.977	<0.0001^b^
LDL-C (mmol/L)		2.445 ± 0.576	2.806 ± 0.938	0.090^a^	3.138 ± 1.179	0.005^a^	3.495 ± 0.712	<0.0001^a^
HDL-C (mmol/L)		1.433 ± 0.214	1.352 ± 0.586	0.011^b^	1.124 ± 0.450	<0.0001^b^	1.157 ± 0.365	<0.0001^b^
γ-GT (U/L)		20.616 ± 5.519	47.650 ± 35.979	<0.0001^b^	47.499 ± 29.959	<0.0001^b^	44.397 ± 27.850	<0.0001^b^
ALT (U/L)		21.447 ± 24.221	34.517 ± 25.530	<0.0001^b^	32.261 ± 23.242	0.005^b^	35.885 ± 26.338	0.001^b^
AST (U/L)		31.757 ± 64.083	25.834 ± 16.840	0.533^b^	26.293 ± 11.281	0.042^b^	24.242 ± 12.751	0.451^b^
ALB (g/L)		47.430 ± 4.162	87.315 ± 94.116	0.262^b^	44.161 ± 7.434	0.002^b^	42.422 ± 5.077	0.0003^b^

Data were presented as mean ± standard deviation or *n* (%).

a Unpaired *t*-test, compared with Control group.

b Mann-Whitney U test, compared with Control group. Abbreviations: BW, body weight; BMI, body mass index; WC, waist circumference; HC, hip circumference; TG, total triglyceride; TC: total cholesterol; LDL-C: low density lipoprotein cholesterol; HDL: high density lipoprotein cholesterol; γ-GT: γ-glutamyl transpeptidase; ALT: alanine aminotransferase; AST: aspartate aminotransferase; ALB, albumin.

c χ2 test, compared with Control group.

### Gut Microbiota Characteristics of Patients With Overweight and Obesity With Dyslipidemia in Xinjiang

**FIGURE 2:**
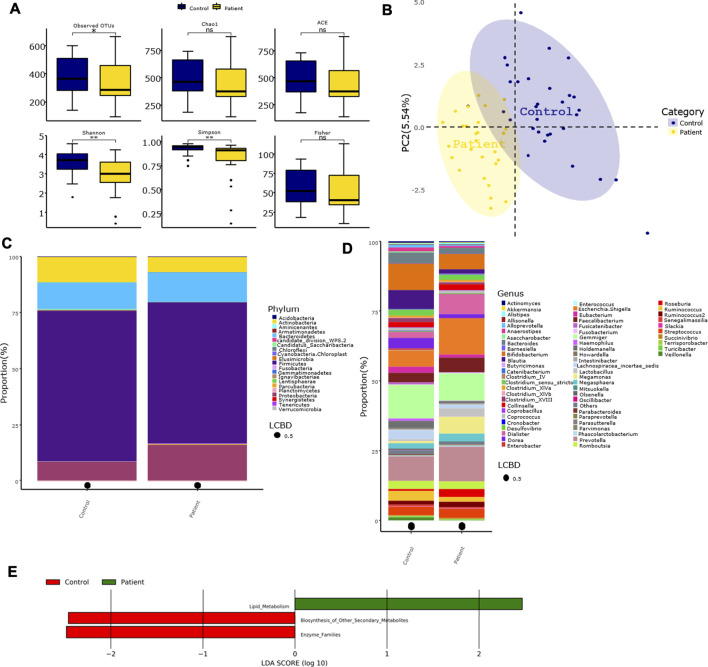
Characteristics of gut microbiota in patients with overweight and obesity with dyslipidemia. **(A)** Alpha diversity index between groups. **(B)** Beta-diversity was visualized by PLS-DA analysis at genus level. Each point represents one individual sample, and points with the same color are in one group. **(C,D)** Bar plot of gut microbiota composition at phylum and genus level. LCBD: Local contributions to beta diversity, LCBD values represent the degree of uniqueness of the sampling units in terms of community composition. **(E)** LDA scores for differentially abundant predicted microbial pathways and classified functional categories (Log10 LDA scores >2) in two groups. Control group: *N* = 31, Patient group: *N* = 27. Asterisk indicated that the differences were statistically significant, one for *p* < 0.05, two for *p* < 0.01, three for *p* < 0.001, four for *p* < 0.0001, ns, not significant.

The differences in the Chao1, ACE, and Fisher’s indices between the control and patient groups were not significant. However, the observed OTU number and the Shannon and Simpson indices were significantly higher in the control group compared with the patient group (Figure 2A). Furthermore, the PLS-DA at the genus level showed that samples in the two groups were obviously and respectively clustered into two different areas (Figure 2B). At the phylum level, the four predominant phyla in the control group were as follows: *Firmicutes*, *Bacteroidetes*, *Actinobacteria*, and *Proteobacteria*, and the four predominant phyla in the patient group were as follows: *Firmicutes*, *Proteobacteria*, *Bacteroidetes*, and *Actinobacteria* (Figure 2C). However, the relative abundance of *Actinobacteria* in the patient group was significantly lower than that of the control group (Table 2). There were no significant differences in the ratio of *Firmicutes/Bacteriodetes* (F/B) between the two groups (Supplementary Figure S1A). At the genus level, the five predominant genera in the control group were as follows: *Gemmiger*, *Bifidobacterium*, *Prevotella*, *Blautia*, and *Escherichia/Shigella*, and the five predominant genera in the patient group were as follows: *Escherichia/Shigella*, *Prevotella*, *Gemmiger*, *Dialister*, and *Megamonas* (Figure 2D). The relative abundance of *Anaerostipes*, *Blautia*, *Bacteroides*, *Bifidobacterium*, *Dorea*, *Veillonella, Lachnospiracea incertae sedis*, and *Ruminococcus* in the patient group was significantly lower than that of the control group, while the relative abundance of *Lactobacillus* was significantly higher than that of the control group. At the species level, the relative abundance of *Bacteroides fragilis* was significantly lower in the patient group vs. the control group (Table 2). Furthermore, the Lefse analysis showed that the functional prediction of the gut microbiota community in the patient group was characterized by lipid metabolism, while that in the control group featured enzyme families and biosynthesis of other secondary metabolites (Figure 2E).

**TABLE 2:** Significant different taxa of participants between control and patient group.

Taxonomy	Species	Control group (mean, %)	Patient group (mean, %)	change	*P*-value^a^
Phylum	*Actinobacteria*	11.327	7.495	Down	0.002
Genus	*Anaerostipes*	1.307	0.570	Down	0.038
	*Bacteroides*	3.656	2.090	Down	0.018
	*Bifidobacterium*	8.752	7.762	Down	0.049
	*Blautia*	6.424	1.934	Down	0.000
	*Dorea*	3.701	1.630	Down	0.003
	*Lachnospiracea_incertae_sedis*	3.160	1.197	Down	0.002
	*Lactobacillus*	0.788	2.678	Up	0.016
	*Ruminococcus*	3.309	1.432	Down	0.028
	*Veillonella*	1.014	0.328	Down	0.002
Species	*Bacteroides_fragilis*	0.826	0.160	Down	0.029
	*Blautia_wexlerae*	5.980	1.606	Down	0.001
	*Dorea_formicigenerans*	2.110	0.813	Down	0.004
	*Eubacterium_hallii*	1.646	0.577	Down	0.000
	*Haemophilus_parainfluenzae*	1.039	0.313	Down	0.002
	*Ruminococcus_bromii*	3.528	1.395	Down	0.038
	*Veillonella_dispar*	1.479	0.428	Down	0.007

Species that were not statistically different between control and patient group and the significant different species with relative abundance <1% were not listed.

a Wilicox test.

### Enterotypes and SCFAs Profiles of Patients With Overweight and Obesity With Dyslipidemia in Xinjiang

**FIGURE 3:**
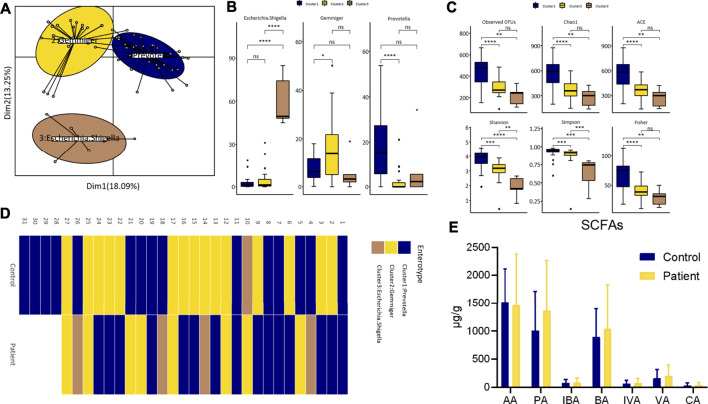
Differences of enterotypes and SCFAs between healthy participants and patients. **(A)** Three clusters were obtained by PCoA analysis at genus level. **(B)** The comparison of the relative abundance of core genus between clusters by wilcox test. **(C)** The comparison of alpha diversity indices between clusters. **(D)** Heatmap of individual’s enterotype. **(E)** Fecal SCFAs level between groups. AA, Acetate; PA, Propionate; IBA, Isobutyrate; BA, Butyrate; IVA, Isovalerate; VA, Valerate; CA, Caproate.

As shown in Figures 3A-C, the enterotypes of the participants in control and patient group were classified into three types, and the alpha diversity of the three enterotypes was as follows: *Prevotella*-type (P-type), *Gemmiger*-type (G-type), and *Escherichia/Shigella*-type (E-type). The individual enterotypes in the control and patient groups are respectively shown in Figure 3D. However, the proportion of the three enterotypes in the two groups was not significantly different (control vs. patient group: 48.4 vs. 55.6% for the P-type, 48.4 vs. 29.6% for the G-type, and 3.2 vs. 14.8% for the E-type; *p* value = 0.159). Although the dominant enterotypes in the two groups were the P-type and G-type, the G-type was more common in the control group, while the E-type was more common in the patient group. However, the levels of the seven types of SCFAs were similar between the two groups (Figure 3E).

### Clinical Efficacy and Gut Microbiota Alterations in the Tested Patients After Orlistat Intervention

**FIGURE 4:**
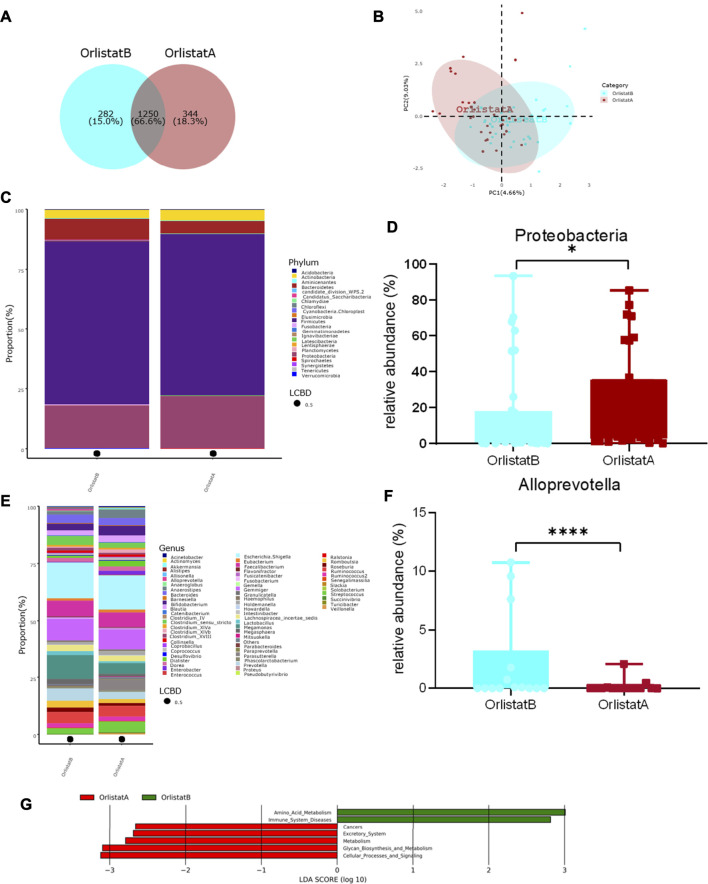
Alterations of gut microbiota before and after orlistat intervention. **(A)** Venn diagram of shared OTUs before and after orlistat intervention. Numbers represent the number of shared OTUs, the percentage represents the proportion of shared OTUs to total OTUs. **(B)** Beta-diversity was visualized by PLS-DA analysis at family level. **(C,D)** Bar plot of gut microbiota composition at phylum level and the relative abundance of *Proteobacteria* was significantly increased after intervention by Metastas analysis. *p < 0.05. **(E,F)** Bar plot of gut microbiota composition at genus level and the relative abundance of *Alloprevotella* was significantly decreased after intervention by Metastas analysis. ****p < 0.0001. **(G)** LDA scores for differentially abundant predicted microbial pathways and classified functional categories (Log10 LDA scores >2) before and after intervention. OrlistatB, before intervention, *n* = 32. OrlistatA, after intervention, *n* = 32.

Compared with the measurements performed before the intervention, the BW, BMI, HC, TC, LDL-C, HDL-C, γ-GT, ALT, AST, and ALB of the tested patients were not significantly altered after the 12-wk course of orlistat. In contrast, the WC, FBG, and TG were significantly decreased after the intervention (Table 3). There were no significant changes in the alpha diversity of gut microbiota between before and after the intervention (Supplementary Figure S1B). The Venn diagram presented in Figure 4A suggests that 66.6% of the OTUs were shared between before and after the intervention, whereas 18.3% of the OTUs were uniquely detected after the intervention. The PLS-DA at the family level showed two different clusters discriminated by the two axes, with some overlapped areas (Figure 4B). At the phylum level, the four predominant phyla were the same before and after inter-vention, i.e., *Firmicutes*, *Proteobacteria*, *Bacteroidetes*, and *Actinobacteria*, and the relative abundance of *Proteobacteria* was significantly increased after the intervention (Figures 4C,D). There was no significant difference in the F/B ratio between before and after the intervention (Supplementary Figure S1A). At the genus level, the five predominant genera before the intervention were as follows: *Escherichia/Shigella*, *Megamonas*, *Gemmiger*, *Faecalibacterium*, and *Prevotella*, while the five predominant genera were as follows: *Escherichia/Shigella*, *Gemmiger*, *Faecalibacterium*, *Parasutterella*, and *Ruminococcus*, and the relative abundance of *Alloprevotella* was significantly decreased, after the intervention (Figures 4E,F). Furthermore, the Lefse analysis demonstrated that the functional prediction of gut microbiota before the intervention featured amino acid metabolism and immune system diseases, while that performed after the intervention was characterized by cancers, excretory system, metabolism, glycan biosynthesis and metabolism, and cellular processes and signaling (Figure 4G).

**TABLE 3:** Changes in patients’ characteristics before and after orlistat or ezetimibe intervention.

Characteristics	Orlistat			Ezetimibe		
	Before intervention	After intervention	*p*-value	Before intervention	After intervention	*p*-value
BW (kg)	80.574 ± 11.574	80.058 ± 11.484	0.551^a^	84.108 ± 15.508	83.561 ± 14.652	0.198^a^
BMI (kg/m^2^)	28.938 ± 3.618	28.805 ± 4.011	0.701^a^	30.272 ± 4.117	30.099 ± 3.919	0.250^a^
WC (cm)	98.604 ± 11.926	96.944 ± 11.216	0.021^a^	101.942 ± 11.935	101.163 ± 11.567	0.147^a^
HC (cm)	105.059 ± 8.549	104.666 ± 8.385	0.180^a^	107.577 ± 10.221	107.094 ± 9.192	0.527^a^
FBG (mmol/L)	5.440 ± 2.306	5.001 ± 1.434	0.013^b^	5.865 ± 2.194	5.668 ± 1.942	0.869^b^
TG (mmol/L)	3.809 ± 2.467	3.127 ± 2.645	0.028^b^	3.578 ± 2.568	2.455 ± 1.688	0.005^b^
TC (mmol/L)	4.941 ± 1.718	4.762 ± 1.001	0.515^a^	5.277 ± 0.977	4.509 ± 0.833	<0.0001^a^
LDL-C (mmol/L)	3.138 ± 1.179	3.197 ± 0.802	0.740^a^	3.495 ± 0.712	3.133 ± 0.616	0.007^a^
HDL-C (mmol/L)	1.124 ± 0.450	1.126 ± 0.231	0.565^b^	1.157 ± 0.365	1.165 ± 0.314	0.903^b^
γ-GT (U/L)	47.499 ± 29.959	52.318 ± 32.968	0.750^b^	44.397 ± 27.850	46.301 ± 29.281	0.819^b^
ALT (U/L)	32.261 ± 23.242	30.303 ± 23.396	0.491^b^	35.885 ± 26.338	30.957 ± 19.018	0.603^b^
AST (U/L)	26.293 ± 11.281	24.796 ± 13.974	0.237^b^	24.242 ± 12.751	22.601 ± 9.846	0.486^a^
ALB (g/L)	44.161 ± 7.434	41.656 ± 5.258	0.072^b^	42.422 ± 5.077	42.704 ± 6.598	0.476^b^

Data were presented as mean ± standard deviation or *n* (%).

a Paired *t*-test.

b Wilcoxon matched pairs signed rank test.

### Enterotype, SCFAs, and Core Taxa Changes in the Tested Patients After the Orlistat Intervention

The enterotypes in the tested patients before and after the orlistat intervention were G-type and E-type. All alpha diversity indices of patients whose enterotype was determined as G-type were significantly higher than those of patients who were typed as E-type (Supplementary Figure S2A). The overall distribution of enterotypes was similar before and after the intervention. The enterotypes of seven individuals were changed after taking orlistat: three of them were changed from G-type to E-type, whereas four were changed from E-type to G-type (Supplementary Figure S2B). Compared with the observation performed before the intervention, the fecal PA levels showed a tendency to decrease after the intervention (*p*-value = 0.08). However, there were no significant differences in the levels of the other fecal SCFAs between before and after the intervention (Supplementary Figure S2C). Six modules correlated with the orlistat intervention were detected in the WGCNA, of which the blue module was the most prominent (*r* = 0.44, *p* = 7.6e−05, Supplementary Figure S3A). Therefore, the hub OTUs in the blue module were annotated and the relative abundance of these genera before and after the intervention are shown in Supplementary Figure S3B and Supplementary Table S1.

### Correlations Between SCFAs, Biochemical Indices, and Orlistat-responsive Core Microbes

**FIGURE 5:**
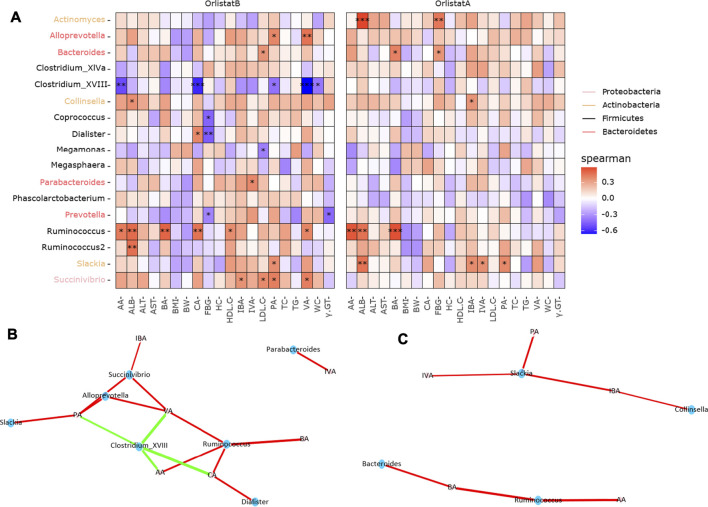
Correlations between orlistat responsive core microbes and biochemical indicators. **(A)** Heatmap of the correlations between core taxa and biochemical data. Asterisk indicated that the correlations were statistically significant, one for *p* < 0.05, two for *p* < 0.01, three for *p* < 0.001. **(B,C)** Network correlations between microbes and SCFAs before and after orlistat intervention. Green indicated the negative correlations, while red indicated the positive correlations.

However, we found a significant correlation between these core microbes, SCFAs, and biochemical parameters (Figure 5A). Before the intervention, *Collinsella*, *Ruminococcus*, and *Ruminococcus*2 were positively correlated with serum ALB; *Coprococcus*/*Dialister* and *Prevotella* were negatively correlated with FBG; *Megamonas* was negatively correlated with LDL-C; *Bacteroides* and *Succinivibrio* were positively correlated with LDL-C; *Clostridium XVIII* was negatively correlated with WC; and *Prevotella* was negatively correlated with γ-GT. The networks of correlation between positive and negative responders to SCFAs indicated that *Clostridium XVIII* was the only negative responder, whereas *Alloprevotella*, *Dialister*, *Parabacteroides*, *Ruminococcus*, *Slackia*, and *Succinivibrio* were positive responders to SCFAs (Figure 5B). After the intervention, *Actinomyces* was positively correlated with serum ALB and FBG; *Ruminococcus* and *Ruminococcus*2 were positively correlated with serum ALB; and *Bacteroides* was positively correlated with FBG. Finally, *Bacteroides*, *Collinsella*, *Slackia*, and *Ruminococcus* were positive responders to SCFAs (Figure 5C).

### Clinical Efficacy and gut Microbiota Alterations in the Tested Patients After the Ezetimibe Intervention

**FIGURE 6:**
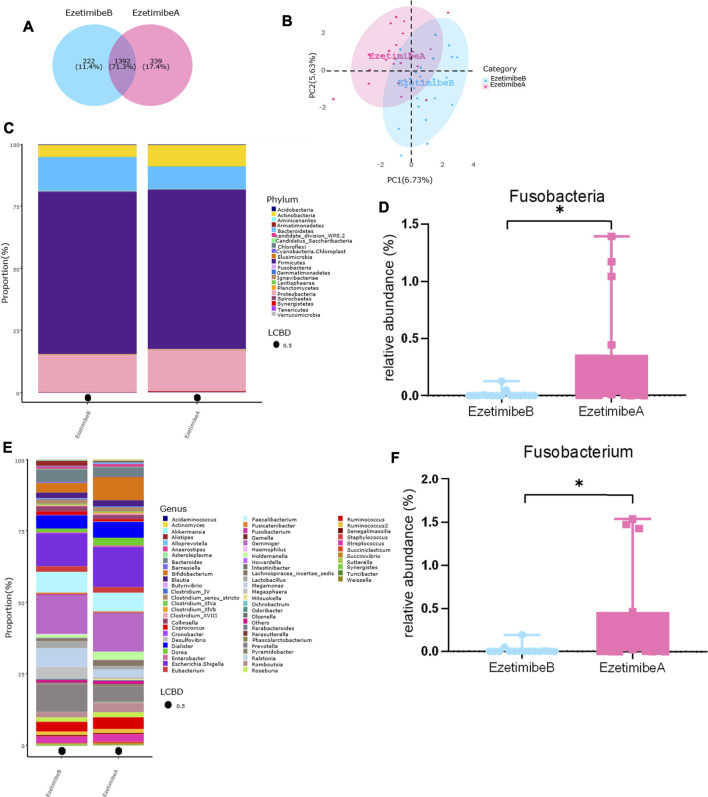
Alterations of gut microbiota before and after ezetimibe intervention. **(A)** Venn diagram of shared OTUs before and after ezetimibe intervention. **(B)** Beta-diversity was visualized by PLS-DA analysis at genus level. **(C**–**F)** Bar plot of gut microbiota composition at phylum and genus level, and the relative abundance of *Fusobacteria* and *Fusobacterium* were significantly increased after intervention by Metastas analysis. *p < 0.05. EzetimibeB, before intervention, *n* = 32. EzetimibeA, after intervention, *n* = 25. One sample was excluded for the poor sequencing.

Compared with that observed before the intervention, the TG, TC, and LDL-C levels in the tested patients were significantly decreased after the 12-wk ezetimibe course. However, there were no significant differences in BW, BMI, WC, HC, HDL-C, γ-GT, ALT, AST, and ALB in the tested patients between before and after the intervention (Table 3). The Venn diagram presented in Figure 6A showed that 71.3% of the OTUs were shared before and after the intervention, while 17.4% of the OTUs were uniquely detected after the intervention. No significant differences in the alpha diversity of gut microbiota were found between before and after the intervention (Supplementary Figure S1B). The PLS-DA at the family level showed that samples from the two different groups were respectively gathered into two clusters (Figure 6B). At the phylum level, the top four phyla were the same regardless of the type of intervention, i.e., *Firmicutes*, *Proteobacteria*, *Bacteroidetes*, and *Actinobacteria*, and no obvious changes in the F/B ratio were found between before and after the intervention (Figure 6C and Supplementary Figure S1A). However, after the intervention, the relative abundance of *Fusobacteria* was significantly increased (Figure 6D). At the genus level, before the intervention, the top five genera were as follows: *Gemmiger*, *Escherichia/Shigella*, *Prevotella*, *Faecalibacterium*, and *Megamonas*. After the intervention, the top five genera were as follows: *Escherichia/Shigella*, *Gemmiger*, *Bifidobacterium*, *Faecalibacterium*, and *Dialister*, and the relative abundance of *Fusobacterium* was significantly increased (Figures 6E,F). No significant alterations in the functional prediction of the gut microbiota community were noted between before and after the intervention (Supplementary Figure S1C).

### Enterotype, SCFAs, and Core Taxa Changes in the Tested Patients After the Ezetimibe Intervention

*Gemmiger* and *Escherichia/Shigella* were the two clusters that were determined as enterotypes in the tested patients before and after taking ezetimibe. No significant differences in the observed OTUs, Chao 1, ACE, and Fisher index were found between the two enterotypes. However, the Shannon and Simpson indices of the G-type were significantly higher than those of the E-type (Supplementary Figure S4A). Similar to the orlistat intervention, the overall distribution of enterotypes was not significantly altered between before and after the intervention. The enterotypes of seven individuals were changed after taking ezetimibe: five of them were changed from G-type to E-type, and two were changed from E-type to G-type (Supplementary Figure S4B). Moreover, the fecal SCFAs were not significantly altered after taking ezetimibe (Supplementary Figure S4C). Six modules correlated with the ezetimibe intervention were detected in the WGCNA, of which the yellow module was the most significant (*r* = 0.57, *p* = 1.4e−07, Supplementary Figure S5A). The hub OTUs in the yellow module were annotated and the relative abundance of these genera are shown in Supplementary Figure S5B and Supplementary Table S2.

### Correlation Between SCFAs, Biochemical Indices, and Ezetimibe-responsive Microbes

**FIGURE 7:**
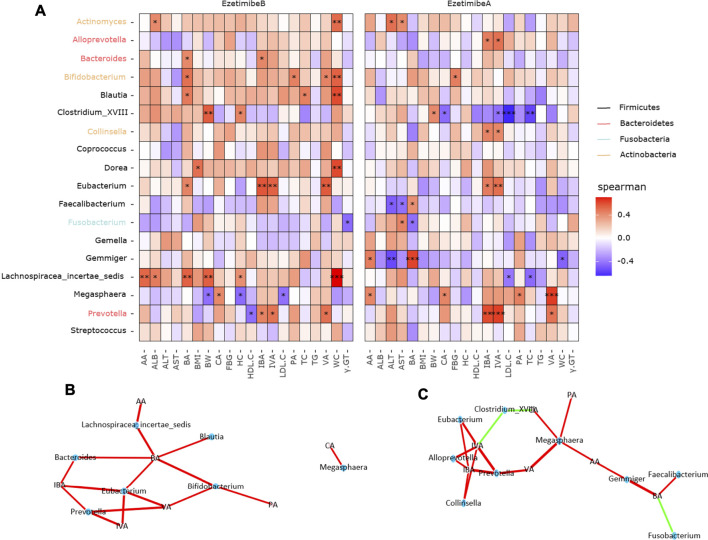
Correlations between ezetimibe responsive core microbes and biochemical indicators. **(A)** Heatmap of the correlations between core taxa and biochemical data. Asterisk indicated that the correlations were statistically significant, one for *p* < 0.05, two for *p* < 0.01, three for *p* < 0.001. **(B,C)** Network correlations of microbes and SCFAs before and after ezetimibe intervention. Green indicated the negative correlations, while red indicated the positive correlations.

Before the intervention, *Actinomyces* was positively correlated with ALB and WC; *Bifidobacterium* was positively correlated with WC; *Blautia* was positively correlated with TC and WC; *Clostridium XVIII* was positively correlated with BW and HC; *Dorea* was positively correlated with BMI and WC; *Fusobacterium* was negatively correlated with γ-GT; *Lachnospiracea incertae sedis* was positively correlated with ALB, WC, BW, and HC; *Megasphaera* was negatively correlated with BW, HC, and LDL-C; and *Prevotella* was negatively correlated with HDL-C (Figure 7A). In addition, *Lachnospiracea incertae sedis*, *Blautia*, *Bifidobacterium*, *Megasphaera*, *Bacteroides*, *Eubacterium*, and *Prevotella* were positive responders to SCFAs (Figure 7B). After the intervention, *Actinomyces* was positively correlated with ALT and AST; *Bifidobacterium* was positively correlated with FBG; *Clostridium XVIII* was positively correlated with BW and negatively correlated with LDL-C and TC; *Faecalibacterium* was negatively correlated with ALT and AST; *Fusobacterium* was positively correlated with ALT; *Gemmiger* was negatively correlated with ALT and WC; and *Lachnospiracea incertae sedis* was negatively correlated with LDL-C and TC (Figure 7A). *Clostridium XVIII* and *Fusobacterium* were negative responders to SCFAs, while *Alloprevotella*, *Eubacterium*, *Megasphaera*, *Gemmiger*, *Collinsella*, *Faecalibacterium*, and *Prevotella* were positive responders to SCFAs (Figure 7C).

## Discussion and Conclusion

The use of germ-free animals and microbiota transplantation showed that the gut microbiota may play a causal role in the development of obesity and associated metabolic disorders. Differences in gut microbiota composition, functional genes, and metabolic activities between overweight/obese and normal-weight individuals were investigated in the Xinjiang region of China, and the microbial responses to antiobesity medications were examined in the present study.

Most studies in this field have found that the gut microbiota of obese people is characterized by an increase in the F/B ratio, the phylum *Firmicutes*, and a decrease in *Bacteroidetes* (Gomes et al., 2018). However, studies focusing on childhood obesity have reported controversial data about the F/B ratio and the proportion of *Firmicutes* and *Bacteroidetes*, which implied that F/B might be a rough tool to predict or examine the gut microbiota associated with obesity (Taylor, 2017). Furthermore, a cross-over study of 29 adults did not find differences in the proportion of *Firmicutes* and *Bacteroidetes* between obese and non-obese individuals using FISH (Duncan et al., 2008). No significant differences in *Firmicutes* to *Bacteroides* ratio was observed between healthy lean and overweight or obese whose dietary intakes and physical activity levels were comparable in a cohort study (Fernandes et al., 2014). Consistently, in the present study, no significant changes in the F/B ratio and the relative abundance of *Firmicutes* and *Bacteroidetes* were found between overweight/obese and normal-weight adults. *Clostridium* and *Faecalibacterium prausnitzii* were suggested as classical SCFAs producers and immunomodulators by many studies (Atarashi et al., 2013; Zhou et al., 2018). Previous works have demonstrated that *Clostridium* species are positively correlated with obesity, while the fecal *Faecalibacterium prausnitzii* amount was not significantly different between obese and lean subjects (Collado et al., 2008; Feng et al., 2014). *Akkermansia muciniphila* was a promising candidate among the various next-generation beneficial microbes, because it was inversely associated with obesity, metabolic disease, cardiovascular diseases, and even cancer (Cani and de Vos, 2017). A recent study found that *Akkermansia muciniphila* abundance was lower in cases with severe obesity, while other studies reported that not only overweight pregnant women, but also their infants, had a higher abundance of *Akkermansia muciniphila* than did normal-weight women and their infants (Collado et al., 2010; Dao et al., 2019). However, in the present study, no obvious changes in the abundance of *Clostridium*, *Faecalibacterium prausnitzii*, and *Akkermansia muciniphila* were found between normal-weight and overweight/obese adults, which might result from the regional and dietary differences of the Xinjiang population compared with those of other studies.

Conversely, in the present study, the microbiota composition of overweight and obese adults with dyslipidemia in Xinjiang was characterized by an overrepresentation of *Lactobacillus* and a decrease in the alpha diversity index and a reduction of *Actinobacteria*, *Bacteroides*, *Bifidobacterium*, and *Bacteroides fragilis*. Conversely, a significant increase in microbial diversity in overweight adults was found in a Japanese population using next-generation sequencing (Kasai et al., 2015). The microbial diversity was normally negatively related to the disease occurrence in adulthood, while the inverse relationship was detected in infancy and childhood because of the unstable state of gut microbiota during early life. Long et al. found that the phylum *Actinobacteria*, particularly the genera *Mobiluncus*, *Corynebacterium* and *Bifidobacterium*, was less abundant among non-diabetic obese subjects compared with normal-weight individuals (Long et al., 2017). Furthermore, research data suggest that a high concentration of *Bifidobacterium* during both infancy and adulthood protects against obesity, which was consistent with the results of our study (Kalliomäki et al., 2008; Collado et al., 2010). *Bifidobacterium* species may decrease fat absorption through the deconjugation of bile acids (Begley et al., 2006), which is possibly related to its reduction in obese subjects. Polysaccharide A of *Bacteroides fragilis*, which belongs to the genus *Bacteroides*, has been highlighted as directing microbiota and host interactions, such as anti-inflammation, and may enhance the efficacy of cancer therapies based on immune checkpoint inhibitors (Erturk-Hasdemir and Kasper, 2018). However, in contrast with the results of our study, Scheepers et al. demonstrated that *Bacteroides fragilis* was present at high concentrations in obese and overweight compared with lean infants or children and was positively correlated with the BMI Z-score (Scheepers et al., 2015). Furthermore, a recent clinical trial of overweight adults on a vegan diet suggested that a smaller reduction in *Bacteroides fragilis* was associated with a greater loss of body weight, fat mass, and visceral fat, and a greater increase in insulin sensitivity (Kahleova et al., 2020). Other studies demonstrated that *Lactobacillus* was positively correlated with weight gain, and a significantly higher *Lactobacillus* concentration was detected both in obese children and adults compared with lean control individuals (Armougom et al., 2009; Bervoets et al., 2013; Million et al., 2013), which was consistent with the results of the present study. These significantly altered microbes, all of which were significantly decreased in obese people, with the exception of *Lactobacillus*, were commonly considered to benefit the health status of the host. Therefore, it is suggested that the gut microbiota characteristics of overweight and obese people with dyslipidemia in Xinjiang were the obvious reduction of species diversity and of some potential beneficial microbes (such as *Bifidobacterium*) without the enrichment of opportunistic pathogens. These results also suggest that the gut microbiota composition of overweight and obese adults with dyslipidemia in Xinjiang, PR China, was mostly different from those reported previously and has its own characteristics. Despite this, the functional prediction of these distinctive microbiota still featured lipid metabolism, which was related to the metabolic phenotypes. This might be the consequence of the study subjects included in the present study, who were characterized by an abnormally elevated BMI and blood lipids vs. the subjects who had only a high BMI enrolled in other studies.

To explore further the gut microbial patterns and metabolic activities between obese and normal-weight individuals in Xinjiang, we also analyzed the enterotypes and SCFAs. Enterotypes were first proposed by Arumugam et al., in 2011 and were determined as clusters dominated by *Prevotella* (P-type), *Bacteroides* (B-type), and *Ruminococcus* (R-type) (Arumugam et al., 2011). The enterotypes were shown to be independent of age, gender, cultural background, and geographical location. Regardless of the geographical region, the P-type and B-type were more robust compared with the third type (i.e. *Bifidobacterium, Faecalibacterium*, and *Ruminococcus*) in the healthy human gut microbiome (Mobeen et al., 2018). Furthermore, these enterotypes have been found and involved in many diseases, especially in obesity (Christensen et al., 2018). For instance, the P-type, B-type, and R-type have been investigated in adults with severe obesity in France (Dao et al., 2019). However, a recent study performed in Mexico found that most normal-weight children had a gut bacterial community dominated by the R-type, while most obese children had a community dominated by the P-type (Maya-Lucas et al., 2019). In the present study, the enterotypes were P-type, G-type, and E-type, which were significantly different from the recent studies regardless of a lean or obese status.

Previous studies indicated that it is likely that differences in diets worldwide have created subgroups of enterotypes (Nishijima et al., 2016). A *Bacteroides* subtype with high abundance of *Bifidobacterium* is prevalent in Japan (Nakayama et al., 2015). The P-type was associated with a diet high in fiber and resistant starch, while the B-type was associated with a diet high in fats and low in fiber (Wu et al., 2011). A recent study reported that a high intake of carbohydrates and fat and a low intake of micronutrients, dietary fiber, and high-quality protein were the characteristics of the current diet in Xinjiang (Yin et al., 2020), which does not present the nutritional structure of a balanced diet. Therefore, we believe that the G-type and E-type found in Xinjiang might associated with a diet high in carbohydrates and fat and low in fiber and protein. Despite this, the existence and generalizability of these proposed enterotype subgroups warrant further investigation using a larger sample size, and more-refined enterotype predictors that model complex bacterial taxa distributions may be needed.

Many studies indicated that SCFAs levels are significantly different between overweight/obese and lean people (Canfora et al., 2015). However, a non-significant change in microbial metabolic activities characterized by a comparable SCFAs level was found in the present study. Bacterial species from the genera *Bacteroides* and *Prevotella* produce acetate and propionate, and those from *Lactobacillus* and *Bifidobacterium* genera mainly produce lactate and acetate (Koh et al., 2016). In the present study, although the abundance of these microbes was different between obese and lean individuals, the ability of these microbes to produce SCFAs was similar, which might be correlated with the relatively stable enterotypes found among these participants, especially those with the P-type.

Antiobesity medication may modulate the gut microbiota, thereby altering fecal SCFAs composition, which may improve metabolic health. Gu et al. demonstrated that acarbose increases the relative abundances of *Lactobacillus* and *Bifidobacterium* in the gut microbiota and depletes *Bacteroides*, which may beneficially affect metabolism in patients with T2D by changing the relative abundance of microbial genes involved in BA metabolism (Gu et al., 2017). Orlistat, as a classical antiobesity agent, was approved by the FDA in the US in 1999 based on its efficacy in the treatment of obesity (Heck et al., 2000). In the present study, the WC, TG, and FBG of patients with overweight/obesity with dyslipidemia decreased significantly after taking orlistat, which indicated that the drug was effective in improving metabolic health, as previously demonstrated, albeit without weight reduction.

However, the use of orlistat has been associated with several mild-to-moderate gastrointestinal adverse effects, such as oily stools, diarrhea, abdominal pain, and fecal spotting, which were related to the increased fat malabsorption (Filippatos et al., 2008). However, whether these adverse effects play roles in modulating the gut microbiota and its metabolites remains unclear. In the present study, the microbial diversity, predominant bacteria, enterotypes, and fecal SCFAs were not significantly altered by a 12-wk course of orlistat. These results were consistent with those of Morales et al., who performed an orlistat intervention study in 10 healthy subjects over 7 days (Morales et al., 2016). However, a recent study demonstrated that the microbiota of HFD-fed mice supplemented with orlistat were characterized by significantly increased *Actinobacteria*, *Proteobacteria*, *Pseudomonas*, *Rhodococcus*, *Roseburia*, and *Acetivibrio*, and significantly decreased microbial diversity, *Firmicutes*, and *Deferribacteres* (Ke et al., 2020). Nevertheless, in the present study, some bacterial populations with a low relative abundance were modified by orlistat, presenting as increased *Proteobacteria* and decreased *Alloprevotella*. *Proteobacteria* is a small-proportion phylum comprising many endotoxin-producing bacteria that might be pathogenic to the host, such as *E.coli*. A recent study found that a HFD diet promoted the flourishment of *Proteobacteria*, while *Ganoderma lucidum* mycelium significantly reversed this effect (Chang et al., 2015). Species of *Alloprevotella*, which belongs to *Bacteroidetes*, could produce moderate amounts of acetic acid and major amounts of succinic acid as end products of fermentation (Downes et al., 2013). We also found that the positively significant correlations between *Alloprevotella* and SCFAs became insignificant with the reduction of *Alloprevotella* caused by orlistat. Animal studies found that both the HFD and high-sucrose diet reduced the abundance of *Alloprevotella*, while the combination of the abrownin and the high-sugar diet promoted the reproduction of *Alloprevotella* (Kong et al., 2019; Yue et al., 2019). These results indicate that the colonic accumulation of dietary fat might result in the enrichment of the opportunistic pathogens with the depletion of potential beneficial microbes related to obesity. Furthermore, previous studies have found that the fecal bile acid level of obese subjects was slightly decreased or kept stable after the administration of orlistat, implying a lower potential for modulating microbiota composition (Trouillot et al., 2001; Ahnen et al., 2007). Here, the absence of significant changes in microbial metabolite levels after the orlistat intervention probably reflected the fact that only small changes were observed in the bacterial populations.

As mentioned above, in the present study, FBG, TG, and WC were significantly decreased by orlistat. Before the orlistat intervention, *Coprococcus*, *Dialister*, and *Prevotella* were negative modulators of FBG in overweight and obese patients with dyslipidemia; however, this correlation became insignificant with the slight increases in *Coprococcus* and *Dialister*, and the modest decreases of *Prevotella* that occurred after the intervention. In contrast, after the intervention, *Actinomyces* and *Bacteroides* were positively correlated with FBG, together with the slight decrease in two genera. A recent study reported that the genus *Actinomyces* showed a strong inverse association with the risk of diabetes (Long et al., 2017). In patients with obesity with metabolic disorders, *Bacteroides* has been found to be negatively correlated with BMI, WC, and LDL-C, whereas *Coprococcus* has been found to be negatively correlated with DBP, TG, and UA, and positively correlated with HDL-C and eGFR (Zeng et al., 2019). These results indicate that these microbes might be keystones that are associated with the hypoglycemic efficacy of orlistat, especially in relation to the abundance of *Actinomyces* and *Bacteroides*, which implies that the depletion of these genera might help orlistat to improve glucose metabolism. An animal study demonstrated that the enrichment of *Pseudomonas*, *Rhodococcus*, *Roseburia*, and *Acetivibrio* in HFD-fed mice supplemented with orlistat was positively correlated with metabolic pathways, including glucose, lipid metabolism, and inflammation pathways (Ke et al., 2020), suggesting that orlistat exerts beneficial effects on glycolipid metabolism by modifying gut microbiota, which may offer a novel mechanism of orlistat action. However, there was no significant correlation between TG, TC, and these core selected genera, regardless of the intervention. The significant correlations between HDL-C, LDL-C, and core microbes detected before the intervention became insignificant after the orlistat intervention. In addition, *Clostridium XVIII* was significantly negatively correlated with WC and SCFAs before the intervention; in contrast, this correlation was not statistically significant regarding the decrease in *Clostridium XVIII* afforded by orlistat. *Clostridium XVIII* is one of the SCFAs producers and was believed to induce Treg production, thereby participating in the anti-inflammation response of the host (Atarashi et al., 2013). These results indicate that the improvement in the lipid metabolism afforded by orlistat might be the consequence of the malabsorption of dietary fat, regardless of the modification of these selected microbes and SCFAs, although the network correlations between SCFAs and core taxa were dynamically altered by orlistat. Therefore, the benefits of glucose metabolism from orlistat were related to the reduction of microbes that responded to orlistat, especially *Actinomyces* and *Bacteroides*; however, a similar phenomenon was not found in lipid metabolism.

Hypolipidemic drugs are prescribed in most of cases for the treatment of overweight and obesity accompanied by dyslipidemia. Several studies have shown that the gut microbiota is able to regulate the host cholesterol metabolism (Wahlström et al., 2016; Kriaa et al., 2019; Le Roy et al., 2019). In the present study, not surprisingly, the serum TG, TC, and LDL-C were significantly decreased by ezetimibe, which is a widely approved hypolipidemic drug. Similar to orlistat, the alpha diversity, predominant bacteria, enterotypes, and SCFAs were not significantly altered by ezetimibe. Consistently, no change in total or major bacteria, despite a selective significant increase in *Lactobacillus* spp., was found in normocholesterolemic mice treated with ezetimibe (Catry et al., 2015).However, in the present study, bacteria with low abundance, presenting as *Fusobacteria* and *Fusobacterium*, rather than *Lactobacillus*, were significantly increased after ezetimibe therapy. Furthermore, the significant increase in the abundance of *Fusobacterium* afforded by ezetimibe was positively correlated with AST and negatively correlated with butyric acid. A previous study stated that *Fusobacterium* was responsible for bile acid desulfation (Robben et al., 1989), which further indicated the possible links between *Fusobacterium* and liver dysfunction. Previous evidence suggested a potential active role for *Fusobacterium* (*Fusobacteria*), specifically *F. nucleatum*, in colorectal cancer (Hussan et al., 2017). Our previous study found that *Fusobacteria* and *Fusobacterium* were decreased by the potential probiotic strain *Bifidobacterium bifidum* TMC3115 in middle-aged and elderly individuals, while the TC and LDL-C were also significantly reduced by TMC3115 (Wang et al., 2019). Therefore, these results indicate that the colonic accumulation of dietary cholesterol results in a slight flourishment of *Fusobacterium*, which has a low abundance and is potentially harmful to the host. Findings from an animal study suggested that the *Lactobacillus* spp., which are negatively correlated with the expression of genes related to cholesterol metabolism, were significantly increased by ezetimibe (Ke et al., 2020). Zhong et al. suggested that ezetimibe is more effective when microbiota is absent and speculated that this was because ezetimibe may be co-administered with antibiotics and/or probiotics clinically (Zhong et al., 2015). No studies have examined the effects of ezetimibe on gut microbiota and its metabolites in patients with metabolic disorders. To the best of our knowledge, the present study was the first analysis focusing on this topic, and detailed studies are required to address the gut microbiota alterations afforded by ezetimibe in the future.

Before the ezetimibe intervention, *Dorea*, *Clostridium XVIII* and *Lachnospiracea incertae sedis* were positively correlated with BW, BMI, HC, and WC, respectively. Conversely, *Megasphaera* was negatively correlated with BW and HC. In contrast, these correlations were not observed in patients enrolled in the orlistat intervention group, indicating that the microbial responses are dependent on the specificity of the medications. In patients with obesity with metabolic abnormalities, it was reported that *Dorea* was positively correlated with BMI, WC, TC, and LDL-C, while *Blautia* was positively correlated with WC, TC, and LDL-C (Zeng et al., 2019), which was partly consistent with the results of the present study. These genera were also predominant in the obesity group and were shared among patients with different metabolic disorders (Zeng et al., 2019). As an acetate producer, *Blautia* can drive insulin release and promote metabolic syndromes, such as hypertriglyceridemia, fatty liver, and insulin resistance (Zhang et al., 2015). Moreover, *Dorea* was negatively associated with insulin resistance (Brahe et al., 2015). However, a recent study reported that *Megasphaera* was associated with overweight and obesity (Kaplan et al., 2019). These results suggest that the relationship between gut microbiota and metabolic indicators in subjects who have overweight and obesity accompanied by dyslipidemia, such as those enrolled in the present study, was partly distinct from that detected in individuals who had a high BMI exclusively. After the intervention, although TG levels decreased significantly, no significant correlations with core taxa were found. In contrast, TC and LDL-C were negatively correlated with *Clostridium XVIII* and *Lachnospiracea incertae sedis*, while these two genera were slightly altered by ezetimibe. Recently, new bacterial phylotypes belonging to *Lachnospiraceae* have been associated with a high coprostanol level in healthy humans (Antharam et al., 2016). It is reported that coprostanol is one of the metabolites of cholesterol, and that the production of coprostanol can protect the gastrointestinal tract against the accumulation of cholesterol (Kriaa et al., 2019). *Clostridium* was involved in the various processes of BA metabolism, including deconjugation, oxidation, epimerization, 7-dehydroxylation, and desulfation (Kriaa et al., 2019). The absorption of dietary cholesterol accounts for approximately one-third of the pool of cholesterol in the body, which was inhibited by ezetimibe in this study, resulting in the reduction of serum lipid levels. Bile acids can shape the gut microbiota community by promoting the growth of bile-acid-metabolizing bacteria and inhibiting the growth of other bile-sensitive bacteria through its direct antimicrobial effects or its indirect effects via FXR and the immune system (Wahlström et al., 2016). Although the network correlations between SCFAs and these selected microbes were significantly modified by ezetimibe, the fecal SCFAs contents were not significantly altered, which was in accordance with the findings of the orlistat intervention. Therefore, we speculated that *Clostridium XVIII* and *Lachnospiracea incertae sedis* were ezetimibe-responsive microbes that reduced TC and LDL-C, which suggests the possibility of targeting selected gut microbiota to modulate cholesterol metabolism. However, the underlying mechanisms warrant further detailed study in the context of BA metabolism.

In conclusion, the gut microbial characteristics of patients with overweight and obesity with dyslipidemia in Xinjiang were partly distinct from those reported for subjects who had a high BMI exclusively. In addition, the particularity of the Xinjiang population indicated that the contribution of the gut microbiota to obesity should be analyzed by adapting to local conditions. The relatively stable gut microbiota and SCFAs recorded between before and after orlistat/ezetimibe intervention suggested the limited microbial modifications of anti-obesity medicines. The significant decreases of TG by orlistat and ezetimibe may not associate with the altered microbes but because of the medication induced malabsorption of lipid. Despite this, lowering effect of FBG was more sensitive in orlistat intervention, *Actinomyces* and *Bacteroides* might be the assistants in improving host glucose metabolism, while the reduction of TC and LDL-C were more effective in the ezetimibe intervention, *Clostridium XVIII* and *Lachnospiracea incertae sedis* might be targets for modulating the host cholesterol metabolism. These findings may provide scientific evidence of the precise prevention and control of metabolic diseases (obesity, etc.) in Xinjiang.

This study has four limitations: ① we did not recruit the age-matched healthy controls with the patients in the two drug intervention groups, which result in the sample size was relative small when compare the gut microbiota between control and patient group. ② we did not collect the dietary information which might influence the clinical efficacy of orlistat and ezetimibe through gut microbiota or conduct a randomized controlled trial to ensure the dietary intake in control. ③ we did not detect the bile acids (BAs), one of the mainly metabolites of gut microbiota, which was reported its association with metabolic disorders (including obesity). Since the patients had liver dysfunction and the enterohepatic circulation of BAs might be obstructed, something new might be found if we further detect the BAs. ④ the dose effects of the two drugs on gut microbiota and its metabolites were not considered because of the clinical trial, and animal study or *in vitro* fermentation should be considered.

## Data Availability

The data presented in the study are deposited in the Sequenced Read Archive (SRA) repository, accession number (PRJNA67844).
