# Supplementary material for: Distinctive Gut Microbiota in Patients with Overweight and Obesity with Dyslipidemia and its Responses to Long-term Orlistat and Ezetimibe Intervention: A Randomized Controlled Open-label Trial
Source: Front Pharmacol. 2021 Aug 26;12:732541. doi: 10.3389/fphar.2021.732541 (PMC8426637; doi:10.3389/fphar.2021.732541)
Supplement: Supplementary file 1 [file DataSheet1.PDF]

Supplementary Material

1 Supplementary Figures and Tables

1.1 Supplementary Figures

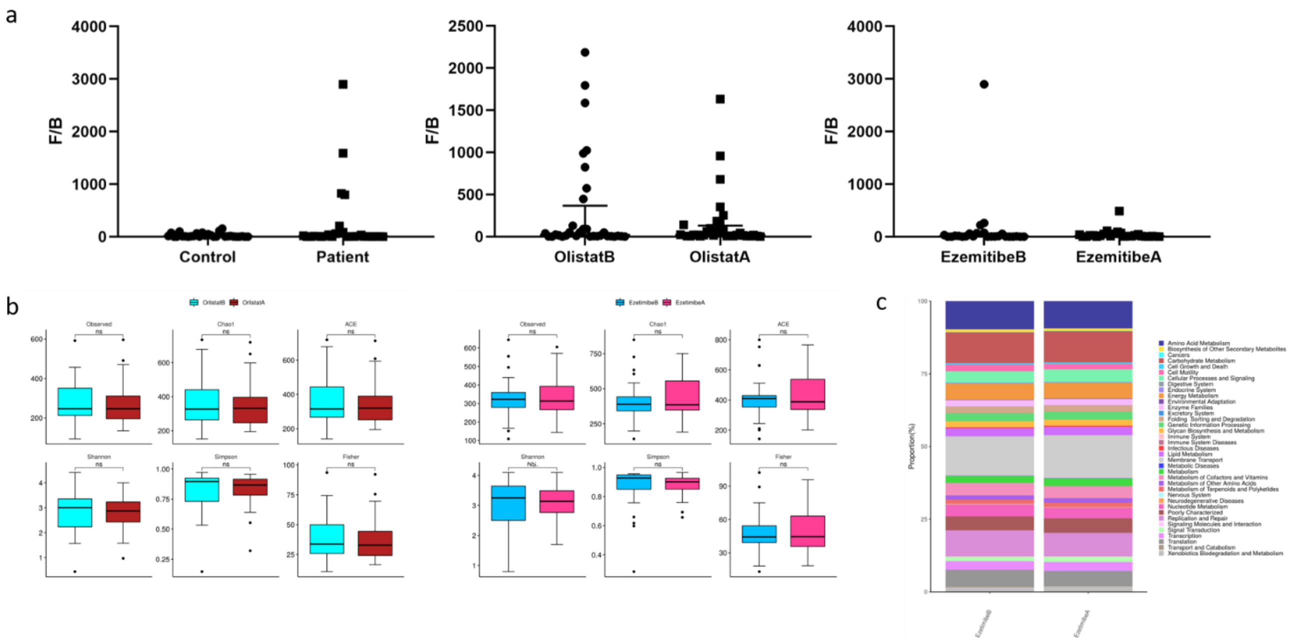

Supplementary Figure 1. Gut microbiota diversity and F/B. a) alpha diversity index before and after orlistat/ezetimibe intervention. b) *Firmicutes*/*Bacteroidetes* ratio. c) Proportions of KEGG pathways at level 2 encoded in the microbiome in two groups. No significant differences were found in these comparisons between groups. ns: not significant.

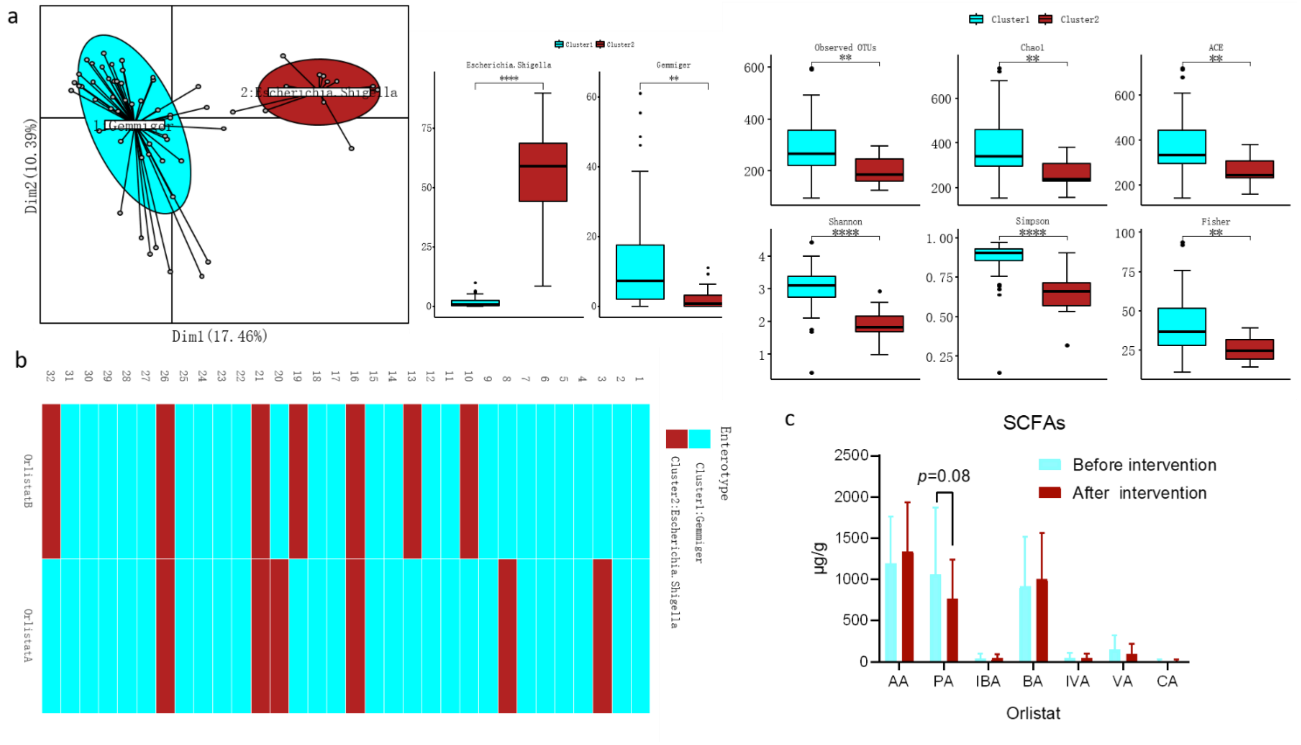

Supplementary Figure 2.Differences of Enterotypes and SCFAs before and after orlistat intervention. a) Determinations of enterotypes between groups. b) Heatmap of individual's enterotype. C) Fecal SCFAs level presented as mean ± SD.

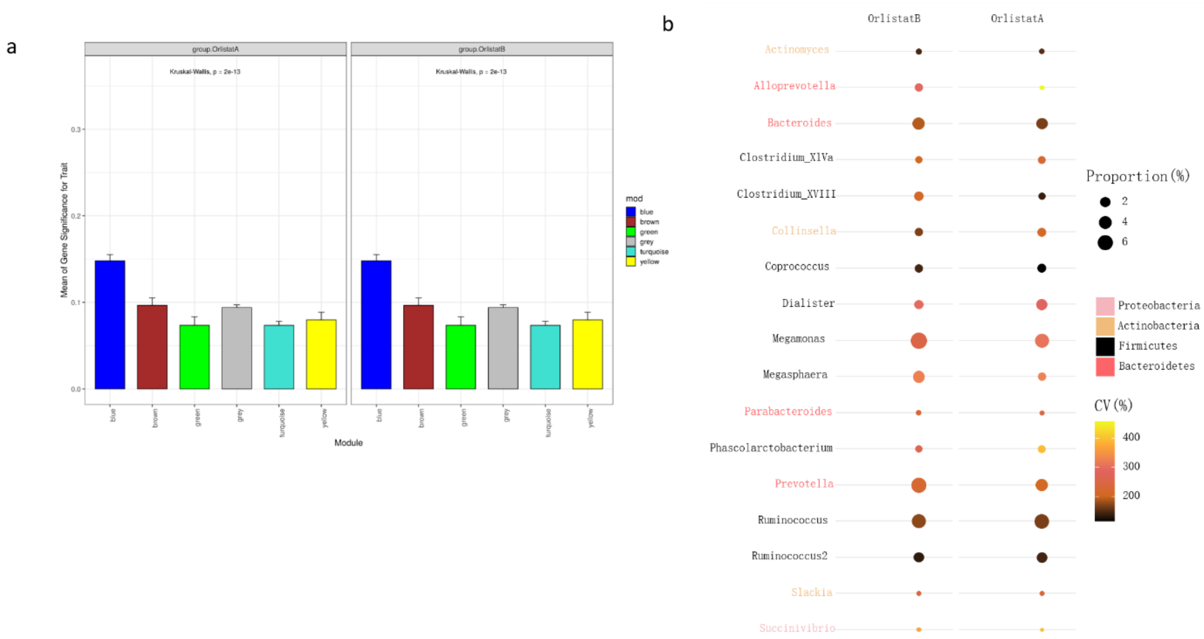

Supplementary Figure 3.Core taxa responded to orlistat by WGCNA. a) Modules which were significantly related to orlistat. b) Mean relative abundance of core taxa in blue module before and after intervention.

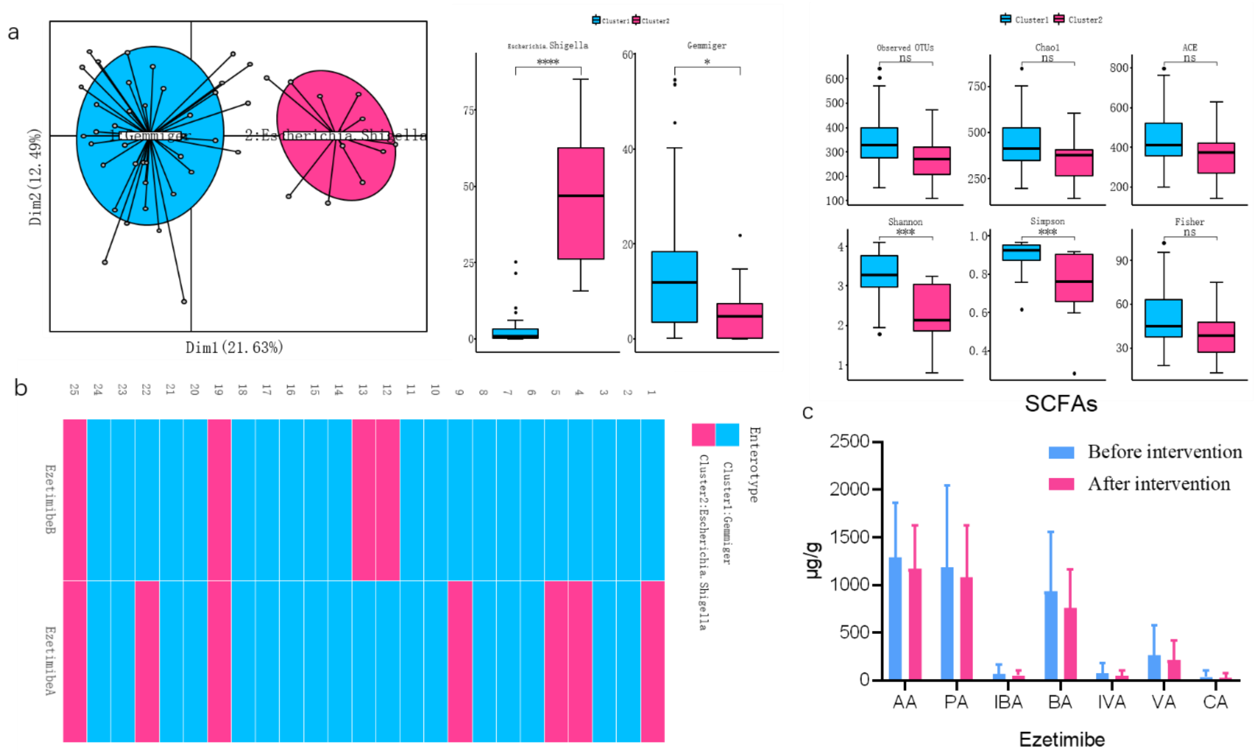

Supplementary Figure 4. Differences of Enterotypes and SCFAs before and after ezetimibe intervention. a) Determinations of enterotypes between groups. b) Heatmap of individual's enterotype. C) Fecal SCFAs level were presented as mean ± SD.

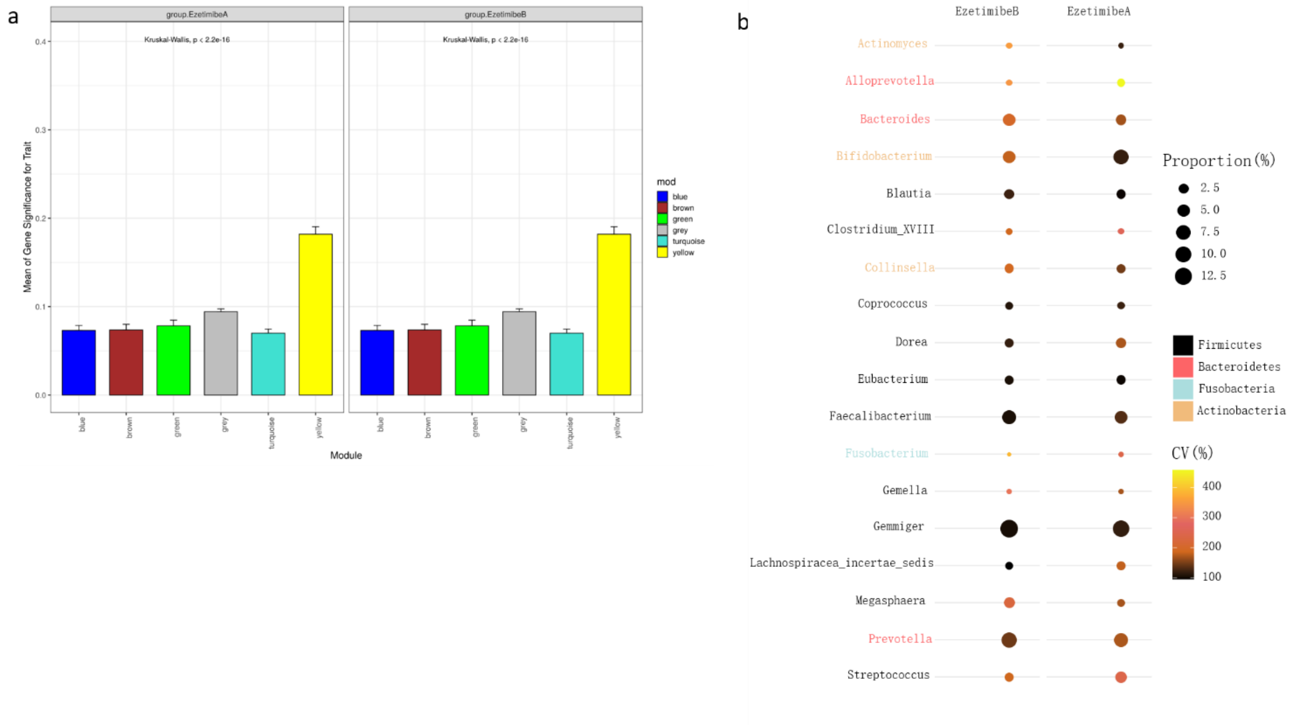

Supplementary Figure 5. Core taxa responded to ezetimibe by WGCNA. a) Modules which were significantly related to ezetimibe. b) Mean relative abundance of core taxa in yellow module before and after intervention.

1.2 Supplementary Tables

Table S1 Mean relative abundance of core microbes responded to orlistat

Genus	mean. OrlistatB.	variance. OrlistatB.	stderr. OrlistatB.	mean. OrlistatA	variance. OrlistatA	stderr. OrlistatA	*p*-value	*q*-value	change
Actinomyces	0.240325762	1.20607E-05	0.000613918	0.180754423	7.35274E-06	0.000479346	0.790297874	1	down
Alloprevotella	0.956975051	0.000774411	0.004919385	0.081624493	1.39167E-05	0.000659468	7.21563E-07	9.71E-05	down
Bacteroides	3.347874589	0.003990421	0.011166944	2.752561823	0.002057618	0.008018764	0.705294705	1	down
Clostridium_XlVa	0.640695275	0.000166197	0.002278961	0.813617878	0.000328752	0.003205233	0.558608306	1	up
Clostridium_XVIII	1.717257995	0.001303505	0.006382361	0.693491377	8.89328E-05	0.001667078	0.133866134	1	down
Collinsella	1.027259084	0.000292369	0.003022671	1.316996106	0.000750828	0.004843901	0.633366633	1	up
Coprococcus	1.011989239	0.000205985	0.002537129	1.328121729	0.000227957	0.002669018	0.410589411	1	up
Dialister	1.400717999	0.001675593	0.007236178	2.537457014	0.005112468	0.012639803	0.456543457	1	up
Megamonas	7.418658632	0.033551229	0.032380177	5.036436819	0.023675471	0.027200339	0.579420579	1	down
Megasphaera	3.303173669	0.011243199	0.018744331	1.159465845	0.001399895	0.00661413	0.323676324	1	down
Parabacteroides	0.210408684	2.272E-05	0.000842615	0.12177018	8.61504E-06	0.000518864	0.548475261	1	down
Phascolarctobacterium	0.607129873	0.000242568	0.002753228	0.841726384	0.001092563	0.005843167	0.300246211	1	up
Prevotella	6.103416406	0.018040311	0.023743625	3.481826397	0.004998668	0.012498334	0.323676324	1	down
Ruminococcus	4.864406958	0.007063878	0.01485753	5.501673284	0.008242883	0.016049614	0.797202797	1	up
Ruminococcus2	2.002285506	0.000697672	0.004669288	2.153480207	0.000927798	0.00538458	0.848151848	1	up
Slackia	0.118082723	7.85739E-06	0.000495523	0.084408315	3.65109E-06	0.000337782	1	1	down
Succinivibrio	0.089797372	1.09762E-05	0.000585667	0.049981999	3.85808E-06	0.000347225	1	1	down

Metastat analysis was used to compare the differences of the relative abundance of species between groups, and $p<0.05$ was considered statistically significant.

Table S2 Mean relative abundance of core microbes responded to ezetimibe

Genus	mean. OrlistatB.	variance. OrlistatB.	stderr. OrlistatB.	mean. OrlistatA	variance. OrlistatA	stderr. OrlistatA	*p*-value	*q*-value	change
Actinomyces	0.399985254	0.00019747	0.002810481	0.245611873	8.33872E-06	0.000577537	0.453764	1	down
Alloprevotella	0.315046258	0.000116233	0.002156225	1.132985865	0.002661345	0.010317646	0.749251	1	up
Bacteroides	5.113960135	0.009854705	0.019854173	3.295887367	0.002940735	0.010845709	0.461538	1	down
Bifidobacterium	4.9527519	0.007783812	0.017645183	8.21440652	0.00911012	0.01908939	0.242757	1	up
Blautia	2.488799716	0.00088149	0.005937981	2.152549283	0.000425435	0.004125216	0.661339	1	down
Clostridium_XVIII	0.402710317	5.45029E-05	0.001476521	0.3727711	9.25865E-05	0.001924437	1	1	down
Collinsella	2.015685547	0.001417033	0.0075287	1.771627421	0.000654658	0.005117256	0.789211	1	down
Coprococcus	1.130068017	0.000146092	0.002417369	0.814224744	9.35041E-05	0.00193395	0.328671	1	down
Dorea	1.608749454	0.000345609	0.003718113	2.792800196	0.002201144	0.009383269	0.27972	1	up
Eubacterium	1.920586909	0.00038411	0.003919746	2.047126974	0.000389529	0.0039473	0.821179	1	up
Faecalibacterium	6.81544584	0.004827889	0.013896602	5.726696996	0.005559265	0.014912096	0.573427	1	down
Fusobacterium	0.010405639	1.59765E-07	7.99412E-05	0.205095371	2.39868E-05	0.000979527	0.062375	1	up
Gemella	0.069442586	4.25531E-06	0.000412568	0.085225258	2.02087E-06	0.000284314	1	1	up
Gemmiger	13.37164956	0.01796322	0.026805388	11.62223634	0.017686009	0.026597751	0.633367	1	down
Lachnospiracea_incertae_sedis	0.938258993	7.68574E-05	0.001753368	1.812262804	0.001034363	0.006432302	0.23976	1	up
Megasphaera	3.297380245	0.005363717	0.01464748	1.008915721	0.000281546	0.003355868	0.142857	1	down
Prevotella	9.544610636	0.018405155	0.02713312	7.720814146	0.016918383	0.026014137	0.639361	1	down
Streptococcus	2.128972592	0.001515052	0.007784733	3.455576312	0.006787768	0.016477582	0.58042	1	up

Metastat analysis was used to compare the differences of the relative abundance of species between groups, and $p<0.05$ was considered statistically significant.
